# The language of gene ontology: a Zipf’s law analysis

**DOI:** 10.1186/1471-2105-13-127

**Published:** 2012-06-07

**Authors:** Leila Ranandeh Kalankesh, Robert Stevens, Andy Brass

**Affiliations:** 1 School of Computer Science, University of Manchester, Oxford Road, Manchester, M13 9PL, UK; 2 Faculty of Life Sciences, University of Manchester, Oxford Road, Manchester, M13 9PL, UK; 3 Current address: Tabriz University of Medical Science, Tabriz, Iran

## Abstract

**Background:**

Most major genome projects and sequence databases provide a GO annotation of their data, either automatically or through human annotators, creating a large corpus of data written in the language of GO. Texts written in natural language show a statistical power law behaviour, Zipf’s law, the exponent of which can provide useful information on the nature of the language being used. We have therefore explored the hypothesis that collections of GO annotations will show similar statistical behaviours to natural language.

**Results:**

Annotations from the Gene Ontology Annotation project were found to follow Zipf’s law. Surprisingly, the measured power law exponents were consistently different between annotation captured using the three GO sub-ontologies in the corpora (function, process and component). On filtering the corpora using GO evidence codes we found that the value of the measured power law exponent responded in a predictable way as a function of the evidence codes used to support the annotation.

**Conclusions:**

Techniques from computational linguistics can provide new insights into the annotation process. GO annotations show similar statistical behaviours to those seen in natural language with measured exponents that provide a signal which correlates with the nature of the evidence codes used to support the annotations, suggesting that the measured exponent might provide a signal regarding the information content of the annotation.

## Background

### The gene ontology and annotation

The Gene Ontology (GO) is used extensively in biology. It provides a structured set of concepts that can be used to describe genes and gene products. These concepts are divided into three separate sub-ontologies focused on molecular function (MF), biological process (BP) and cellular component (CC) [1]. The GO has now been used to annotate many of the standard databases of genes and gene products. This annotation helps to integrate biological resources across various experimental organisms and different data bases [2-4]. The power of the GO annotation is that it allows unambiguous communication of knowledge among biologists as to the functionality of gene products, at the same time as making the biological knowledge computer-comprehensible [3,4]. GO annotation is undertaken either manually, automatically, or by some combination of both [4]. The GO Consortium provide codes that indicate the evidence to support the association between a specific GO term and gene product (for example through sequence similarity or direct experimental support). Evidence codes should not be directly used as a measure of the annotation quality [5]; they can, however, help inform the level of belief a user might have in the GO terms assigned [6].

A number of studies have attempted to address issues of annotation quality, for example by looking at the consistency of coding between different annotators [7]. Another study introduced an Annotation Confidence Scoring system for comparing the annotation of genes and gene products to those found in a reference genome set [8]. Others have used the GO evidence codes and term depth in the GO to provide evidence of quality [9]. There is some evidence that sources annotated through manual curation are of higher quality than those annotated automatically [10] as they are the result of the combined effort of many scientists [11]. None of these methods, however, has addressed the core question of how effective the annotations are in conveying meaning to a wider biological audience. We therefore need methods that determine the extent to which annotation is meeting user requirements. Unfortunately, we have very few ways of judging whether the set of annotations produced to describe a collection of genes/gene products in a database works effectively in communicating knowledge between the annotator and the end user of those annotations.

### Language and the principle of least effort

The GO provides a vocabulary used by annotators to encode information regarding gene product function, information that the wider community then need to decode. The annotation associated with a gene product can be thought of as a sentence made up of words from GO.

It has long been known that natural languages show power-law behaviour. For example Zipf’s law states that for any sufficiently large corpus word frequency is approximately inversely proportional to word rank (in which words are ordered by their frequency within the text, the most common ranked first). Indeed, Zipf’s law is considered as the statistical characteristic of human language [12,13], and as a wider property of many different complex systems [14]. This pattern has even been observed in a number of extinct and undeciphered languages such as Meroitic [15], and in the mysterious encrypted 15th century Voynich manuscript [16].

If N(r) is the number of words in a text with rank r then Zipf’s law can be expressed as:

(1) $$\text{N}\left(\text{r}\right)\sim {\text{r}}^{-\alpha }$$

where α is the Zipf’s law exponent.

There are a number of different ways in which this behaviour can be represented mathematically - power law behaviour, Zipf’s law, Pareto’s law - that can be demonstrated to be equivalent [17]. For example, if P (ƒ) is the proportion of words in a text with frequency ƒ then the power law can also be expressed as:

(2) $$\text{P}\;\left(ƒ\right)\sim {ƒ}^{–\beta }$$

It is straightforward to show that β and α are related by:

(3) $$\beta =1+\frac{1}{\alpha }$$

For typical single author sources in English β is about 2 [18-20]. There can, however, be variations around this value. For example, in the speech of young children β is around 1.6 [21] whereas β > 2 has been found in sets of nouns taken from single author texts [22]. Almost all texts analysed have values of β in the range [1.6-2.4] [23]. Zipf further argued that the power law behaviour arose from a principle of “least effort” in communication. A communication process can be thought of as having three components; a speaker, a listener and a message. The principle of “least effort” examines the work required from the speaker and the listener in communicating a message [12,24].

Similarly, we can view annotation as a process of communication. Consider the process of annotating the cellular location of the gene product integrin alpha8. The simplest annotation for the speaker (annotator) to produce is a frequently used (and ambiguous) term such as “cell” (GO:0002623). Such an annotation would, however, push greater effort on to the person using the annotation – the listener. The listener’s job is easiest if the term used is clear and unambiguous, for example “*integrin complex”* (GO:0008305). This, however, requires significant effort from the speaker in identifying such rarely used GO terms.

### Zipf's law and the gene ontology

In this paper we have applied methods of computational linguistics to large repositories of GO annotation data from a number of complete published genomes. The objectives are to determine the extent to which:

GO annotation from complete genomes show power law behaviour;

the exponent of the power law provides insights into the nature of the underlying annotation;

computational linguistic analysis provide insights into the annotation process.

To do this we have retrieved genome annotations from the Gene Ontology Annotation (GOA) project. In particular, the GOA data can be regarded as a gold-standard annotation set, with a significant portion that has been extensively curated by human experts.

## Methods

Gene Ontology identifiers and evidence codes were retrieved from each of the genome projects covered by the Gene Ontology Annotation (GOA) project (the version published in October 2009). Table 1 shows the data sets that were obtained and the total number of annotations and distinct number of GO identifiers included in each.

**Table 1:** Total number of annotations and the number of distinct GO identifiers for each of the data sets used in the study in terms of three separate sub-ontologies

**Species**	**Sub-Ontology**	**GOA**	
		**Total number of annotations**	**The number of distinct GO IDs**
**Hs**	**CC**	51,640	889
	**MF**	55,781	2,844
	**BP**	58,320	5,259
**Mm**	**CC**	45,933	641
	**MF**	60,919	2,318
	**BP**	59,133	4,239
**Dr**	**CC**	23,179	304
	**MF**	47,651	1,187
	**BP**	34,158	1,513
**Sc**	**CC**	29,563	626
	**MF**	26,292	1,611
	**BP**	31,797	1,963
**Rn**	**CC**	53,342	50
	**MF**	63,050	2,776
	**BP**	74,943	5,411

CC – Cellular Component sub-ontology, MF - Molecular Function sub-ontology and BP - Biological Process sub-ontology. Homo sapiens (Hs), Mus musculus (Mm), Danio rerio (Dr), Saccharomyces cerevisiae (Sc), Rattus norvegicus (Rn).

The mouse and human GOA data sets were then further sub-divided using GO evidence codes to produce eight new data sets with different levels of support from the evidence codes. A set of high confidence (HC) data were derived chosen by selecting annotations labeled with at least one of the IDA (Inferred from Direct Assay); IPI (Inferred from Physical Interaction); IMP (Inferred from Mutant Phenotype ); TAS (traceable Author Statement); EXP (Inferred from experiment); IC (Inferred by Curator); IEP (Inferred for Expression Pattern) or IGI (Inferred from Genetic Interaction) evidence codes. A set of low confidence (LC) data were derived by selecting annotations labeled with IEA (Inferred from Electronic Annotation) evidence codes. These data sets are characterized and described in Table 2.

**Table 2:** The total number of annotations and the number of distinct GO identifiers of each of the Homo sapiens (Hs) and Mus musculus (Mm) data sets in terms of the three separate sub-ontologies by evidence code

**Species**	**Sub-ontology**	**GOA**			
		**High Confidence**		**Low Confidence**	
		**The number of distinct GO IDs**	**Total number of annotations**	**The number of distinct GO IDs**	**Total number of annotations**
**Hs**	**CC**	642	16,744	572	31,164
	**MF**	1,974	20,250	1,735	31,709
	**BP**	3,172	18,594	3,642	33,820
**Mm**	**CC**	487	11,784	232	28,918
	**MF**	1,364	10,467	1,320	45,185
	**BP**	3,846	264,78	731	26,473

It can be difficult to calculate an accurate exponent for the Zipf’s law exponent if the data are presented in the form of a frequency vs rank graph, particularly as the data for high rank (low frequency) terms are often noisy. By representing the data in the form of a Pareto distribution it is possible to measure the exponent much more accurately [17]. This is because the Pareto distribution is expressed in terms of the cumulative distribution frequency:

(4) $$P\left(X\geq x\right)\sim x^{-k}$$

where the distribution shape parameter *k* can be converted to the Zipf’s law exponent α via:

(5) $$\alpha =\frac{1}{k}$$

and to the power law exponent β as below:

(6) β=1+k

The cumulative frequency graph is well defined for all values of x, and removes the problem of noise in the low frequency terms [17].

The data on the GO identifier frequencies were therefore analysed using the Matlab packages plfit, plplot and, plpva (version 1.0.10 published in January 2010) developed by Clauset and Shalizi [25]. These packages attempt to fit a power law model to the empirical data (represented as a Pareto distribution) and determine the extent to which the data can be effectively modeled using a power law. These tools provide two statistics describing the data. The first is a *P*-value that is used to determine the extent to which the power law model is appropriate. If the *P*-value is greater than 0.1 we can regard the power law to be a plausible model of our data. The second statistic produced is β, the exponent of the power law.

## Results

### Annotation and power law behaviour

Some of the most frequently used terms in the annotation data are some of the most generic (low term depth). For example the term GO:0005515 (protein binding) is typically one of the top two most frequent terms in all the MF data analysed and is only two levels down from the root of the molecular function sub-ontology. The top 25% of the most commonly used GO terms for human molecular function have an average depth of 4.6, compared with an average depth of 6.4 for the 25% least commonly used terms. A similar pattern is repeated for all the sub-ontologies in all species examined in this paper (data not shown). This difference is significant (*p* < 0.001 in a paired t-test), demonstrating that the most commonly used terms are typically less specific (higher in the ontology) than those which are used less frequently (deeper in the ontology).

**Figure 1:**
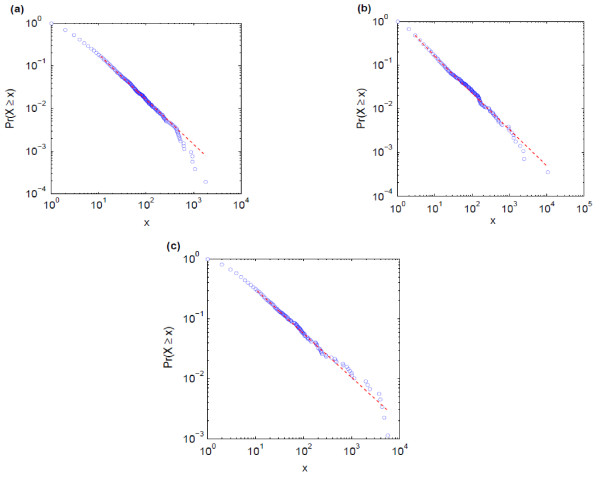
**The cumulative distribution function Pr(x) plotted as a function of frequency (x) for GO gene annotations contained within Human GOA.** The straight line shows the region of the plots for which a power law was found to provide a good model of the data [25]. 1(**a**) Annotation from the biological process sub-ontology, 1(**b**) annotation from the molecular function sub-ontology, and 1 (**c**) annotation from the cellular component sub-ontology The measured power law exponents, β, (were 2.04, 1.83, and 1.73 respectively. For all graphs *p*-value > 0.55, suggesting that the power law does provide a plausible model of the data.

Figure 1 shows the log-log plots of cumulative frequency vs. term rank (Pareto plots) for data from the human GOA. It can be seen from these figures that there is strong support for a power law model for these data for the annotations from all three sub-ontologies, as demonstrated in the *P*-values returned from the fitting software.

Table 3 shows the results obtained for the GOA datasets as defined in Table 1. In all cases the data are well-described by a power law, with exponents in the range observed for language (1.6 < β < 2.4). By examining the results we can see that there are differences between the exponents measured for the BP sub-ontology compared with the CC and MF ontologies; the mean value of β for the GO BP sub-ontology is 2.13, for the MF sub-ontology is 1.81, and for the CC sub-ontology is 1.71. The difference between the mean values from the BP compared with the CC and MF ontologies is significant (*p* < 0.001). There is no significant difference between the exponents measured for the CC and MF sub-ontologies. One interesting anomaly is the value of the exponent measured for the biological process sub-ontology of *D. rerio* at 1.88 compared with the mean of 2.13.

**Table 3:** **Results obtained from the power law analysis of each of the data sets characterized in Table**2

**Species**	**Ontology**	**GOA**	
		***β***	**P-value**
**Hs**	**CC**	1.73	0.63
	**MF**	1.83	0.55
	**BP**	2.04	0.65
**Mm**	**CC**	1.69	0.74
	**MF**	1.76	0.36
	**BP**	2.08	0.97
**Dr**	**CC**	1.62	0.74
	**MF**	1.69	0.91
	**BP**	1.88	0.11
**Sc**	**CC**	1.86	0.29
	**MF**	1.88	0.78
	**BP**	2.27	0.42
**Rn**	**CC**	1.68	0.24
	**MF**	1.91	0.85
	**BP**	2.38	0.76

β is the power law exponent and *P*-value is a statistic used to determine how good a model the power law is of the data. If *P* > 0.1 we can assume that the power law does provide a good description of the data. H. sapiens (Hs), M. musculus (Mm), D. rerio (Dr), S. cerevisiae (Sc), R. norvegicus (Rn).

The analysis was then repeated for the data-sets obtained from the mouse and human GOA data sets divided into high and low confidence evidence codes (the statistics for which are shown in Table 2). These results are shown in Table 4.

**Table 4:** **Results obtained from power law analysis of each of the data sets characterized in Table**2

**Species**	**Ontology**	**GOA**			
		**HC**	**LC**		
		***β***	**P-Value**	***Β***	**P-Value**
**Hs**	**CC**	1.88	**0.37**	1.62	**0.11**
	**MF**	2.05	**0.18**	1.75	**0.16**
	**BP**	2.12	**0.37**	2.04	**0.62**
**Mm**	**CC**	1.9	**0.43**	1.5	**0.71**
	**MF**	2.15	**0.65**	1.67	0.03
	**BP**	2.6	**0.61**	1.62	0.00

β is the power law exponent and P-value is a statistic used to determine how good a model the power law is of the data. Statistically significant values are denoted in bold. The GO evidence codes used to define the high confidence (HC) and low confidence (LC) data sets are described in the materials and methods.

Again there is a clear trend visible in these results, with the low confidence data showing consistently lower exponents than the full data set, with the highest exponents being measured for the filtered high confidence data. A paired t-test analysis of data measured from the high confidence and low confidence data supports the fact that the difference in exponents between these data sets is significant (*p* = 0.01). It is also interesting to note that two of the annotation data sets with lower values of *β* have P-values < 0.1, i.e. cannot be effectively represented by a power law.

**Figure 2:**
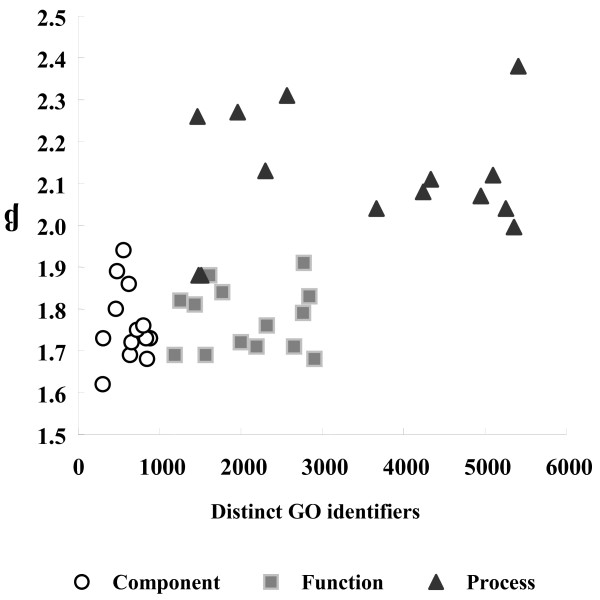
**The power law exponent, β, as a function of the total number of distinct GO identifiers in each of the GO sub-ontologies referenced in table **4** as well as a number of other species datasets taken from Ensembl.**

Using the data from Tables 1 and 3 it is possible to examine β as a function of both the total and distinct number of GO identifiers in each genomic annotation dataset. There is no clear correlation between the size of the data set and the power law exponent (Figure 2). This analysis includes data from a wide range of species data sets from the Ensembl database in addition to the GOA datasets.

## Discussion

We have used computational linguistics methods to examine a range of gene annotation data sets used to populate genome resources. In almost all cases these data sets obey Zipf’s law, with exponents typical of those for human languages (Table 3). The This supports the hypothesis that the GO annotation can be thought of as a language, and that we can think of annotation as a form of communication process with the characteristics of a natural language. This then provides us with a framework in which to look at the effectiveness of the communication process using power law. For example, we have observed a real and significant difference in the power law exponents measured for annotation using the biological process sub-ontology (β ≈ 2.1) compared with that using the molecular function and cellular component sub-ontologies (β ≈ 1.8).

The measured exponent changes in a predictable and significant way as a function of the evidence codes that have been used to support annotation, but not as a function of the size of the annotation available (Figure 2). However, it is not clear that the absolute value of the exponent can be interpreted as a quality measure; for example, we would not want to state that the BP annotations are of higher quality than those done with the MF and CC ontologies. We therefore need to look more deeply into the linkage between the exponent and information transfer. For example, some insights can be drawn from work in statistical mechanics approaches to understanding the behaviour of language [26]. In this work it is hypothesised that the exponent β is proportional to the “temperature” of the communication system, where temperature is to be interpreted as a “willingness to communicate”. This would therefore imply that the increase we see in the value of β as a function of the annotation source (Table 4) reflects an increasing effort in the communication process. Indeed, this observation has been made previously in a number of studies of human language, in which the value of the exponent has been somewhat controversially linked to communication effectiveness [23,24,27,28]. Similarly, there is a large literature (e.g. [29]) which debates the interpretations that can legitimately me be made of the Zipf’s law exponent in linguistics and the extent to which these variations provide insights into communication, whether in whistles between dolphins [30], the nature of the schizophrenic brain [31] or language in children [32]. In particular, much of this analysis has investigated the ways in which differences in language use, communication effectiveness or brain structure are reflected in the measured exponent.

An inference that might therefore be drawn as regards the differences in exponents between the various GO sub-ontologies could therefore be that the information conveyed by BP is fundamentally more complex than that described by the other two sub-ontologies, capturing the processes in which the molecule is involved, rather than a simple molecular function description, or a location in which the activity takes place. That is, we simply have more to say about process than we do about function and cellular location; the biology is more complex in processes. This might intrinsically require more “willingness to communicate” than is needed to describe aspects of molecular function or cellular component. An anomaly in this analysis is the observed low exponent for the *D. rerio* BP sub-ontology, from which we might infer that the information content captured in the annotation for biological processes in this model species is lower than that from the other model organisms (as reflected in the significantly smaller number of published papers on *D. rerio* compared to those of the other model species listed).

One key difference between this analysis and that more generally used in computational linguistics is in the variation of word length. In the GO annotation all words have the same length (the GO Identifier) whereas in natural languages word lengths can vary. A recent paper [33] has revisited one of Zipf’s original observations that word length correlates inversely with frequency [34]. The key finding was that the correlation between word length and information content was better than that between word length and frequency. The analysis presented here, in the rather more controlled environment of genome annotation, has the potential to throw new light on this long-running debate in computational linguistics, as we can separate out the effects of word length and focus specifically on the information content and frequency of terms.

In principle we also believe that the straightforward computational linguistics methods we have applied to GO data in this paper should be more widely applicable to any situation in which data are described using terms from an ontology; for example, medical patient data described using terms from SNOMED-CT [35]. Indeed, we have recently observed very similar Zipf’s law behaviour in a large corpus of primary care general practice data describing patients in Salford (UK) (data not shown).

## Conclusions

In this paper we have demonstrated that computational linguistics, in the form of Zipf’s law, provides a powerful and innovative framework in which to examine GO annotation. As hypothesised, the GO annotation does follow Zipf’s law and there is some evidence that the exponent does provide information on the nature of the annotation; for example, it responds in a predictable way as a function of the evidence codes used to support the annotation. An unexpected finding is that the power law exponent of data described using the process sub-ontology is significantly different to that measured for data in the function and component ontologies. We do not know whether this difference is some fundamental feature of the structure of the GO sub-ontologies, the nature of the biology being communicated, or whether it reflects thought processes in the annotation teams. Such an understanding could be useful in helping improve the use of ontologies for annotation.

While other studies have focussed on consistency or depth of annotation for assessing the quality of annotation [7-9], no study has explored the nature of the annotation from the perspective of the communication of information. The method should provide a straightforward technique for assessing corpora described using terms from ontology in areas beyond just biology and bioinformatics.

## Competing interests

There are no competing interests to this work.

## Authors' contributions

LRK carried out analysis work, participated in the study design and drafted the manuscript. RS participated in the study design and helped to draft the manuscript. AB conceived of the study, participated in its design and coordination and helped to draft the manuscript. All authors read and approved the final manuscript.

## References

[B1] Ashburner M Ball CA Blake JA Botstein D Butler H Cherry JM Davis AP Dolinski K Dwight SS Eppig J Gene Ontology: tool for the unification of biology Nat Genet 2000 25 1 25 29 10.1038/75556 10802651 PMC3037419

[B2] Camon E Magrane M Barrell D Lee V Dimmer E Maslen J Binns D Harte N Lopez R Apweiler R The Gene Ontology Annotation (GOA) Database: sharing knowledge in Uniprot with Gene Ontology Nucleic Acids Res 2004 32 Database issue D262 266 14681408 10.1093/nar/gkh021 PMC308756

[B3] Harris MA Clark J Ireland A Lomax J Ashburner M Foulger R Eilbeck K Lewis S Marshal B The Gene Ontology (GO) database and informatics resource Nucleic Acids Res 2004 32 Database issue D258 D261 14681407 10.1093/nar/gkh036 PMC308770

[B4] Rhee SY Wood V Dolinski K Draghici S Use and misuse of Gene Ontology annotations Nat Rev Genet 2008 9 7 509 515 10.1038/nrg2363 18475267

[B5] Guide to GO Evidence Codes[http://www.geneontology.org/go.evidence.shtml]

[B6] Gross A Hartung M Kirsten T Rahm E Paton NW, Missier P, Hedeler C Estimating the Quality of Ontology-Based Annotation by considering Evolutionary Changes DILS 2009 2009 Berlin Heidelberg: Springer-Verlag 81 87

[B7] Dolan M Ni L Camon E Blake JA A procedure for assessing GO annotation consistency Bioinformatics 2005 21 Suppl 1 i136 i143 10.1093/bioinformatics/bti1019 15961450

[B8] Yang Y Gilbert D Kim S Annotation confidence score for genome annotation: a genome comparison approach Bioinformatics 2010 26 1 22 29 10.1093/bioinformatics/btp613 19855104

[B9] Buza TJ McCarthy FM Wang N Bridge SM Burgess SC Gene Ontology annotation quality analysis in model eukaryotes Nucleic Acids Res 2008 36 2 e12 18187504 10.1093/nar/gkm1167 PMC2241866

[B10] Mulas F Curk T Bellazzi R Zupan B Combi C, Shahar Y, Abu-Hanna A On quality of different annotation sources for gene expression analysis Artificial Intelligence in Medicine 2009 2009 Heidelberg: Springer-Verlag Berlin

[B11] Haas BJ Salzberg SL Zhu W Pertea M Allen JE Orvis J White O Buell CR Wortman JR Automated eukaryotic gene structure annotation using EVidenceModeler and the Program to Assemble Spliced Alignments Genome Biol 2008 9 1 R7 10.1186/gb-2008-9-1-r7 18190707 PMC2395244

[B12] Zipf G Human Behavior and the Principle of least effort: Introduction to human Ecology 1949 Oxford: Addison Wesley

[B13] Grzybek P, Köhler R Exact Methods in the study of language and text 2007 Berlin: Walter de Gruyter GmbH & Co

[B14] Manin DY Zipf's Law and Avoidance of Excessive Synonymy Cogn Sci: A Multidisciplinary J 2008 32 7 1075 1098 10.1080/03640210802020003 21585444

[B15] Smith R Investigation of the Zipf-plot of the extinct Meroitic language Glottometrics 2007 15 53 61

[B16] Landini G Evidence of Linguistic structure in the Voynich manuscript using spectral analysis Cryptologia 2001 25 4 275 295 10.1080/0161-110191889932

[B17] Newman M Power laws, Pareto distribution and Zipf's law Contemp Phys 2005 46 5 323 351 10.1080/00107510500052444

[B18] Ferreri Cancho R Sole R Two regimes in the frequency of words and the origins of complex lexicons: Zipf's law revisited J Quant Linguist 2001 8 3 165 173 10.1076/jqul.8.3.165.4101

[B19] Montemurro M Beyond the Zipf-Mandelbrot law in quantitative linguistics Physica A 2001 300 567 578 10.1016/S0378-4371(01)00355-7

[B20] Montemurro M Zanette D Frequency-rank distribution of words in large text samples: phenomenology and the mode Glottometrics 2002 4 87 98

[B21] Piotrowski RG Pashkovskii VE Piotrowski VR Psychiatric linguistic and automatic text processing Automatic Doc Math Linguist 1995 28 5 28 35

[B22] Balasubrahmanyan VK Naranan S Quantitative linguistics and complex system studies J Quant Linguist 1996 3 3 177 228 10.1080/09296179608599629

[B23] Ferreri Cancho R The variation of Zipf's law in human language The European Phys J B2005b 44 249 257 10.1140/epjb/e2005-00121-8

[B24] Ferreri Cancho R Sole R Least Effort and the origins of scaling in human language PNAS 2003 100 3 788 791 10.1073/pnas.0335980100 12540826 PMC298679

[B25] Clauset A Shalizi C Newman M Power law distribution in empirical data SIAM Rev 2009 51 4 661 703 10.1137/070710111

[B26] Kosmidis K Kalampokisa A Argyrakis P Statistical mechanical approach to human language Phys A: Stat Mechanics Appl 2006 366 495 502

[B27] Ferreri Cancho R Decoding least effort and scaling in signal frequency distributions Phys A: Stat Mechanics Appl 2005 345 275 284

[B28] Ferreri Cancho R Zipf's law from a communicative phase transition The European Phys J B 2005 47 3 449 457 10.1140/epjb/e2005-00340-y

[B29] McCowan B Doyle LR Jenkins JM Hanser SF The appropriate use of Zipf’s law in animal communication studies Anim Behav 2005 69 F1 F7 10.1016/j.anbehav.2004.09.002

[B30] Ferreri Cancho R McCowan B A Law of Word Meaning in Dolphin Whistle Types Entropy 2009 11 688 701 10.3390/e11040688

[B31] Ferrer Cancho R When language breaks into pieces A conflict between communication through isolated signals and language Biosystems 2006 84 242 253 10.1016/j.biosystems.2005.12.001 16406253

[B32] Julien Mayor J Plunkett K Vocabulary explosion: are infants full of Zipf? Proceedings of the 32nd Annual Meeting of the Cognitive Science Society 2010 Cognitive Science Society

[B33] Piantadosi ST Tily H Gibson E Word lengths are optimized for efficient communication PNAS 2011 108 9 3526 3529 10.1073/pnas.1012551108 21278332 PMC3048148

[B34] Zipf G The Psychobiology of Language 1936 Routledge, London

[B35] Cornet R de Keizer N Forty years of SNOMED: a literature review BMC Medical Informatics and Decision Making 2008 8 Suppl 1 S2 10.1186/1472-6947-8-S1-S2 19007439 PMC2582789

